# Compliance and Self-Reporting During the COVID-19 Pandemic: A Cross-Cultural Study of Trust and Self-Conscious Emotions in the United States, Italy, and South Korea

**DOI:** 10.3389/fpsyg.2021.565845

**Published:** 2021-03-16

**Authors:** Giovanni A. Travaglino, Chanki Moon

**Affiliations:** ^1^School of Psychology, Keynes College, University of Kent, Canterbury, United Kingdom; ^2^Department of Psychology, School of Social Science, Leeds Beckett University, Leeds, United Kingdom

**Keywords:** horizontal and vertical individualism and collectivism, shame, guilt, trust, COVID-19, pandemic, self-reporting, social-distancing

## Abstract

The coronavirus COVID-19 pandemic is an unprecedented health crisis. Many governments around the world have responded by implementing lockdown measures of various degrees of intensity. To be effective, these measures must rely on citizens’ cooperation. In the present study, we drew samples from the United States (*N* = 597), Italy (*N* = 606), and South Korea (*N* = 693) and examined predictors of compliance with social distancing and intentions to report the infection to both authorities and acquaintances. Data were collected between April 6th and 8th 2020. We investigated the role of cultural orientations of horizontal and vertical individualism and collectivism, self-conscious emotions of shame and guilt related to the infection and trust in the government’s action. Across all countries, vertical collectivism (VC) predicted stronger shame, whereas horizontal collectivism predicted stronger trust in the government. Only in the United States, VC was associated with stronger trust. Stronger feelings of shame predicted lower compliance and intentions to report the infection to both authorities and acquaintances. In contrast, guilt was associated with stronger intentions to report the infection to the authorities. Finally, trust was associated with stronger compliance and intentions to report the infection to the authorities. Unlike Italy and South Korea, the association between trust on compliance was not statistically significant in the United States, implications of the findings, and directions for future research are discussed.

## Introduction

The coronavirus COVID-19 pandemic is an unprecedented health crisis that has forced nearly a third of the world population into lockdown (Kaplan et al., 2020). Lockdowns are “behavioral” (non-pharmaceutical) measures involving forced isolation, movement restrictions, and active government surveillance. These measures effectively slow the virus’s diffusion because they reduce contagion rates (Cowling et al., 2020; Ferguson et al., 2020; Flaxman et al., 2020). Reducing contagion is an important objective, especially in the absence of effective antiviral drugs, vaccines, or widespread population immunity, all still unavailable in the context of the new pandemic.

To be sustained in time, and be more effective, non-pharmaceutical interventions must rely, at least in part, on citizens’ active cooperation with authorities, especially in those countries characterized by democratic political systems. In the present research, we examined critical predictors of cooperation across three different contexts: the United States, Italy, and South Korea. Specifically, we focused on trust toward the government (e.g., Morse et al., 2016), and self-conscious emotions related to the infection (guilt and shame; Finerman and Bennett, 1995). We contribute to the growing psychological literature on COVID-19 (Capraro and Barcelo, 2020, 2021; Lalot et al.,2020a,b; Van Bavel et al.,2020a,b; Yamada et al., 2021) by investigating the associations between these variables and individuals’ compliance with social distancing rules, as well as their intentions to report the infection to both health authorities and acquaintances/friends. Because cultural values may be associated with these variables differently across contexts, we also examined the role of individual-level cultural orientations of vertical and horizontal individualism and collectivism.

## Horizontal and Vertical Individualism and Collectivism

Culture may be defined as shared meaning, shaping individuals’ basic psychological processes and informing their understanding of the world (Triandis, 2001). Two of the most fundamental dimensions of cultural variations are individualism and collectivism (Triandis, 1995; Hofstede, 2001). These values frame individuals’ interpretation of reality, emphasizing the importance of “the individual” or “the collective,” respectively. Because responses to the novel pandemic are likely to involve tradeoffs and adjustments between these two value frameworks, individualism and collectivism are likely to play an essential role in how people behave.

Individualism and collectivism reflect the extent to which cultural groups value independence vs interdependence (Markus and Kitayama, 1991; Kitayama et al., 2009; Park et al., 2016). In individualistic cultures, individuals are socialized toward independence, autonomy of the self and self-reliance. Conversely, in cultures where collectivism is a principal value, individuals are socialized toward interdependence, an interconnected self and the importance of relationships. Individualism and collectivism have attracted a substantial share of research attention across various countries and settings (Gudykunst and Ting-Toomey, 1988; Hofstede, 2001; House et al., 2004).

These dimensions have recently been extended to consider cultures’ different emphasis on equality vs hierarchy (Triandis and Gelfand, 1998; Oyserman et al., 2002; Shavitt et al.,2011a,b). “Horizontal” cultures place importance on equality in status, either in the context of an independent (horizontal individualism, HI) or interdependent (horizontal collectivism, HC) self. Conversely, “vertical” cultures place importance on hierarchical relationships and differences in status, either in the context of competing individuals (vertical individualism, VI) or ranked groups (vertical collectivism, VC). This fourfold typology is an important predictor of a range of behaviors and attitudes (Triandis and Gelfand, 1998; Shavitt et al., 2006,2011a,b; Moon et al., 2018; Travaglino and Moon, 2020).

In the present research, we sampled participants from three different contexts characterized by different prevalent cultural themes. The United States’ dominant themes are individualism and verticality (e.g., Shavitt et al., 2006; Torelli and Shavitt, 2010), with a strong emphasis on the uniqueness and independence of the self as well as status and competition. In Korea, the prevalent cultural themes are group harmony, obedience to authority and an emphasis on status hierarchies, a configuration of values congruent with VC (Triandis and Gelfand, 1998; Shavitt et al., 2006). Relatively less research has examined the Italian context concerning the horizontal and vertical individualism and collectivism (HVIC) typology. However, there seems to be some evidence that the prevalent cultural theme in Italy falls between the United States and Korea in terms of the individualism-collectivism dimension, with a stronger emphasis on horizontality compared to the other two countries (Hofstede et al., 2010; Burton et al., 2019; Germani et al., 2020).

Beyond country-level differences, there is heterogeneity in the values that individuals within countries endorse (cf. Oyserman et al., 2002; Green et al., 2005; Cho et al., 2010; Taras et al., 2016; Burton et al., 2019). It is thus essential to examine endorsement of cultural values at the individual level, as well as variations across contexts. In the subsequent text, “*cultural values*” or “*themes*” refer to the country level of analysis, and “*cultural orientations*” refer to the individual level of analysis.

In the present study, we investigated how cultural orientations within countries characterized by different cultural themes may predict a range of responses to the virus-related emergency, namely trust in the government’s action, and self-conscious emotions of shame and guilt. We then investigated how such factors predict individuals’ intentions to comply with social distancing and report the infection to authorities and acquaintances.

### Trust in Government

Trust in government refers to beliefs and attitudes about the government’s competence and good faith (cf. Levi and Stoker, 2000; Nannestad, 2008). It is a critical feature of the relationship between individuals and institutions. Trust is linked with the government’s performance and reflects the levels of social and civic engagement within society (i.e., social capital; Putnam, 2000). Societies in which the government is efficient and citizens have higher social capital also tend to report stronger trust in government (Tyler, 2001, 2006; Job, 2005; Blind, 2007; Keele, 2007).

The degree of trust in authorities has especially significant implications in emergency and risk situations, where norms about appropriate behavior are unclear, and events may unfold in unpredictable ways. For instance, individuals’ lack of confidence in the governments’ ability to handle terrorist attacks may seriously harm officials’ efforts to shape public responses to such attacks (Wray et al., 2006). Conversely, reliable communication that inspires confidence can help the government reduce anxiety and prevent harm among citizens (Covello, 2003; Wray et al., 2004).

In the health context, trust in authorities was associated with individuals’ compliance with authorities’ recommendations during the H1N1 2009 pandemic in both the United Kingdom (Rubin et al., 2009) and Italy (Prati et al., 2011). Similarly, research from Tang and Wong (2003) showed an association between a composite measure of trust in institutions (including the government’s ability to control the spread of the infection) and the likelihood of wearing a face mask during the outbreak of the Severe Acute Respiratory Syndrome (SARS) in Hong Kong. More recently, Morse et al. (2016) demonstrated that distrust in authorities was associated with reduced usage of health services in Liberia during the Ebola outbreak (2014–2016). In the present study, we tested the role of trust in the governments’ ability to handle the current COVID-19 pandemic by comparing three different countries. We also investigated the cultural values that predicted trust in each of the three settings.

### Self-Conscious Emotions: Guilt and Shame in Response to Infection

Research has yet to examine the role of emotions in individuals’ compliance with authorities’ recommendations in the course of an epidemic outbreak (cf. Prati et al., 2011). Especially relevant in the context of diseases and infections are self-conscious emotions of guilt and shame. Individuals experience guilt or shame when they perceive they have done something wrong, or in response to stigma and blame (Giner-Sorolla, 2012). Although similar, guilt and shame refer to two different appraisals of the self (Lewis, 1971). Individuals feel guilt when they feel responsible for the consequences of a specific action, such as acting in ways that may increase the likelihood of contracting the coronavirus. Instead, shame involves an appraisal of the self as immoral and unworthy (Giner-Sorolla, 2012). Whereas guilt is generally defined as a private emotion, shame is theorized as externally driven (see Benedict, 1946; Wolf et al., 2010). This is because guilt implies a negative evaluation of the self by *oneself*, whereas shame implies a negative assessment of the self by *others*.

Guilt and shame play an essential role in shaping individuals’ health-related decisions in various settings (Donahue and McGuire, 1995; Finerman and Bennett, 1995). These emotions have been examined in the context of multiple conditions, such as sexually transmitted diseases (Goldenberg et al., 2008), cancer (Chapple et al., 2004), type 2 diabetes (Browne et al., 2013), and obesity (Conradt et al., 2008). For instance, concerning sexually transmitted diseases, these emotions are associated with lower disclosure to partners, and lower intentions to seek treatment or testing (Cunningham et al., 2002; Balfe et al., 2010). Moreover, lung cancer patients may perceive guilt and shame about the disease, with significant impact on their intentions to disclose the disease or seek support (e.g., Chapple et al., 2004).

In this research, we investigated the role of guilt and shame in the context of individuals’ responses to the COVID-19 pandemic. Groups may tighten their norms when confronting ecological threats such as outbreaks of diseases (Gelfand et al., 2011; Gelfand et al., 2017). Tighter norms are beneficial because they foster group coordination, sustaining collective efforts (Gelfand et al., 2021). However, tighter norms may also encourage the stigmatization of those perceived as undermining them. For example, across many countries, several social media campaigns stigmatize individuals who were perceived as defying social distancing norms. Moreover, there is anecdotical evidence that contracting the infection leads to the stigmatization of the survivors (Maslin Nir, 2020), and blame toward them (Reicher and Drury, 2021).

### The Present Research

To summarize, this research investigated the role of trust and self-conscious emotions in predicting individuals’ intentions to comply with social distancing norms and to report the infection to either health authorities or acquaintances. We examined these factors in three contexts (the United States, Italy, and South Korea) characterized by different prevalent cultural themes of HI and VI, and HC and VC. Moreover, we examined the role of individuals’ cultural orientations within each country.

Concerning shame and guilt, research differentiating between the two emotions suggest that shame has mostly negative implications and is associated with avoidance and withdrawal, whereas guilt is associated with more positive coping and engagement (Tangney and Fischer, 1995; Tangney, 1998; Conradt et al., 2008). These findings suggest that shame should be associated with lower compliance and intentions to report the infection to authorities and others. In contrast, guilt may have fewer negative implications and even be associated with more substantial compliance and reporting.

We investigated differences in the role of shame and guilt across countries. Research suggests that self-conscious emotions are more prevalent, socially constructive, and have fewer negative implications for individuals’ well-being and behavior in contexts characterized by higher collectivistic values and influenced by Confucianism (Menon and Shweder, 1994; Kitayama et al., 1997; Fischer et al., 1999; Li and Wang, 2004; Wong and Tsai, 2007). This is because these emotions are broadly consistent with the culturally sanctioned goals of self-improvement and adherence to collective standards and norms. In contrast, in individualistic contexts, shame and guilt are seen as negative emotions that should be avoided, and they are thus less socially constructive. This evidence suggests that VC, which emphasizes duties and obligations toward group goals, should predict stronger self-conscious emotions concerning the infection. Conversely, HC, which emphasizes equality and interdependence should predict lower levels of self-conscious emotions. Moreover, such emotions (and particularly shame) should have more substantial negative implications in more individualistic countries where VC is not the dominant value.

Concerning trust, we predicted a positive association between trust toward the government’s efforts to tackle the pandemic and individuals’ intentions to comply with social distancing norms and report the infection to health authorities (e.g., Tang and Wong, 2003; Rubin et al., 2009; Prati et al., 2011; Morse et al., 2016). This is because higher trust in the government means that individuals are more likely to believe in the competence and good faith of the governments’ recommendations, and abide by its regulations (Tyler, 2001).

However, the meaning and implications of trust may be shaped by culture (Shin and Park, 2004; Yuki et al., 2005). Cross-cultural research indicates that the association between trust and individualism and collectivism is complex and multifaceted, owing to the very different operationalizations of these constructs adopted within the literature (see Realo and Allik, 2009). Definitions of individualism that emphasize competition and narrow self-interest (similar to VI) suggest that individualism may have negative implications for trust (Putnam, 2000; Gelfand et al., 2004). However, evidence from cross-country comparisons indicates that individualism (conceptualized as autonomy and self-sufficiency, closer to HI) is associated with stronger (rather than weaker) interpersonal trust (Allik and Realo, 2004; Realo and Allik, 2009). There is also some evidence pointing at the relevance of horizontality and collectivism in fostering the emergence of trust. For instance, Realo et al. (2008) found that countries that scored higher on institutional collectivism – the extent to which institutional arrangements favor collective action (Gelfand et al., 2004) – displayed higher levels of social capital, an essential component of trust in government (Job, 2005).

At the individual level, research by Beilmann and Realo (2012) indicates that social capital is positively associated with two components of collectivism (i.e., relationships with peers, and dedication to the nation), as well as one of individualism (i.e., mature self-responsibility; Job, 2005). Similarly, research shows that the HC orientation – which encompasses values such as benevolence toward others and interdependence – is a predictor of generalized trust (Shin and Park, 2004). In the narrower context of trust toward the authorities, there is also some evidence of an association between VC and authority endorsement in crisis communication (Jakubanecs et al., 2018). This evidence can be explained by the fact that the VC orientation emphasizes respect for authority (see Devos et al., 2002). These findings suggest that both HC and VC may be relevant in predicting trust.

In the analyses, we tested our hypotheses controlling for gender and age due to the samples’ heterogeneity included in the analyses. Moreover, we added political orientation (left-right) as a covariate to control for individuals’ stance toward the current government. Finally, because countries experienced different infection rates, we controlled for individuals’ perceived danger related to the spread of the virus within the country.

## Materials and Methods

### Participants and Procedure

Five-hundred-and-ninety-seven participants were recruited from the United States (296 men, 291 women, 7 other, 3 preferred not to answer; *M*_age_ = 39.35, SD_age_ = 11.62). Of the American participants, 43.6% indicated they lived in a city/large town, 42.4% in a smaller/average town, 14.1% in a village/rural area. Participants were from different states, including Florida (9.7%), New York (8.4%), California (7.7%), Texas (6.5%), Pennsylvania (5.4%), and Ohio (5.4%). The other states represented in the sample each accounted for <5% of the total number of participants.

Six-hundred-and-six participants were recruited from Italy (301 men, 294 women, 8 other, 3 preferred not to answer; *M*_age_ = 26.94, SD_age_ = 7.72). Of the Italian participants, 41.1% indicated they lived in a city/large town, 40.4% smaller/average town, 18.5% village/rural. Participants were from different regions, including Piemonte (21.8%), Lazio (14.7%), Lombardia (10.7%), Veneto (8.9%), Campania (8.7%), Sicilia (6.6%), and Emilia Romagna (5.9%). The other regions represented in the sample each accounted for <5% of the Italian sample.

Six-hundred-and-ninety-three participants were recruited from South Korea (342 men, 346 women, 5 preferred not to answer; *M*_age_ = 44.46, SD_age_ = 13.15). Of the Korean participants 81.8% indicated they lived in city/large town, 16.2% in a smaller/average city, and only 2% in a village/rural area. Participants were from different metropolitan cities and provinces, including Seoul (28.6%), Busan (16.0%), Incheon (14.9%), Daegu (12.4%), Gyungi (8.7%), Gwangju (7.1%), and Daejeon (6.3%). The other regions represented in the sample each accounted for <5% of the Korean sample.

Data from the United States and Italy were collected using Qualtrics via Prolific Academic© (see Peer et al., 2017). Data from Korea were collected using Qualtrics via a local research panel. The studies involving human participants were reviewed and approved by the Psychology Ethics Committee of Leeds Beckett University. Participants were paid 2 GBP in Italy and the United States, and 2.25 GBP in Korea.^1^ All scales included for the present study were initially developed in English and subsequently translated into Italian and Korean. Back-translation was used to achieve equivalent meanings in the two languages following guidelines by Brislin (1986).

Participants were invited to participate in a larger research study on “political and current issues” which also included a module on the “the current COVID-19 situation.” Items were presented to participants in random order. After completing the measures, all participants were debriefed in writing, thanked, and compensated for their time. Data in all countries were collected between April 6th and 8th 2020 (the COVID-19 outbreak was declared a Public Health Emergency of International Concern on January 30, 2020). At that time, the United States had reported 12,895 deaths due to COVID19, Italy 17,129 and Korea 200. According to the University of Oxford’s Stringency index, the three countries had 72.69/100, 91.67/100, and 82.41/100 stringency levels between April 6th and 8th. The Stringency Index is a measure of the severity of the government’s responses to the pandemic. The index is a composite measure of seven indicators rescaled to vary from 0 to 100 (Hale et al., 2020). A higher number means that more restrictions are in place in a given country (see Hale et al., 2020 for details).

### Measures

#### Horizontal and Vertical Individualism and Collectivism

Twelve items drawn from Sivadas et al.’s (2008) 14 item-scale were used to measure HI (3 items; e.g., *I enjoy being unique and different from others in many ways*), VI (3 items; e.g., *I enjoy working in situations involving competition with others*), HC (3 items; e.g., *The well-being of my co-workers is important to me*), and VC (3 items; e.g., *I would do what would please my family, even if I detested that activity*).^2^ Participants answered items using a 7-point Likert scale (*1* = *strongly disagree* to 7 = *strongly agree*). The structure of the short scale of horizontal and VI and collectivism has been validated in four contexts (Sivadas et al., 2008) and further confirmed by a recent cross-cultural study (Moon et al., 2018).

#### Shame and Guilt

Feelings of shame and guilt were measured using one item for each emotion. Participants were asked to indicate the extent to which they would feel ashamed and guilty if they became infected with the new coronavirus COVID-19 (1 = *not at all*, 7 = *very much*).

#### Trust in Governments

Participants indicated the extent to which they agreed or disagreed with a statement “I trust how the government of my country is handling the spread of the coronavirus” using a 7-point scale (1 = *strongly disagree*, 7 = *strongly agree*).

#### Compliance With Social Distancing

Using six items, we measured the extent to which people complied with standard guidelines indicating how to behave during the COVID-19 emergency. The items were “I avoid leaving my home unnecessarily,” “I wash my hands often,” “I follow instructions from health authorities,” “I stay away from crowded places,” “I advise others about how to act in response to the virus,” and “I stand away from others in public places” (1 = *not at all*, 7 = *very much*).

#### Self-Reporting of the Infection to Authorities and Acquaintances/Friends

Participants reported their intentions to report the infection to health authorities and acquaintances/friends. Participants first read “if I suspected I were infected with the new coronavirus…” and answered two items “I would notify the health authorities immediately” “I would hide it from my acquaintances and friends” (1 = *not at all*, 7 = *certainly*).

#### Concerns About the Virus

Using a 7 point scale, participants indicated how concerned they were by the spread of the new coronavirus (COVID-19) in their country (1 = *not at all concerned*, 7 = *extremely concerned*).

#### Political Orientation

Participants indicated how they would describe themselves considering their country’s current political context using a 100-point scale slider (0 = *I am a left-winger*, 100 = *I am a right-winger*). Political orientation was added as a covariate to the model.

### Analytical Strategy and Statistical Power

Data were analyzed using structural equation models with latent and observed variables. Analyses were performed using R with the lavaan (Rosseel, 2012), semTools (Jorgensen et al., 2019), and ccpsych (Fischer and Karl, 2019) packages. The recommended sample size to be able to detect a minimum small-to-medium effect size (δ = 0.2) at 80% power and α = 0.05 in a model with 5 latent and 27 observed variables is *N* = 376 (Soper, 2019).

We first sought to establish measurement invariance across countries for the latent measures included in the final model (He and van de Vijver, 2012; Fischer and Karl, 2019). Measurement invariance indicates that a construct is interpreted similarly by respondents in different groups (i.e., cultures, nations, etc.) and can thus be meaningfully compared. Specifically, using a confirmatory factor analysis and robust standard errors, we tested measurement invariance of the 12-item horizontal and vertical individualism and collectivism (HVIC) scale, and the 6-item compliance scale. For each scale separately, we first tested the configural invariance models to examine whether all items loaded on the respective latent factors across countries. These models were then compared to models where factor loadings were constrained to be equal across countries (i.e., metric invariance). Finally, we fixed intercepts to test for scalar invariance. We sought to establish partial invariance for models that did not achieve full invariance. Partial invariance involves individuating what loading(s) or intercept(s) are causing misfit, thus allowing them to vary freely across the groups compared. Partial invariance (with at least two invariant indicators) is often considered a more realistic and sufficient goal in cross-cultural research (cf. Steinmetz, 2018).

Overall model fit was evaluated using four indices, comparative fit indices (CFI: acceptable ≥0.95, excellent ≥0.97), adjusted goodness of fit index (AGFI; acceptable ≥0.90, excellent ≥0.95), Root Mean Square Error of Approximation (RMSEA; acceptable ≤0.10, excellent ≤0.05) and Standardized Root Mean Square Residual (SRMR; acceptable ≤0.10, excellent ≤0.05; Hu and Bentler, 1998; Schermelleh-Engel et al., 2003). We also report χ^2^, although it should be noted that this index is less reliable due to its dependency on multivariate normality and sample size (Schermelleh-Engel et al., 2003). Invariance was determined by examining whether the CFI difference between the constrained nested models was higher than the recommended threshold of ΔCFI = 0.01 (Cheung and Rensvold, 2002).

Subsequently, to test the hypotheses, we estimated a multi-group structural equation model in which self-conscious emotions of shame and guilt, and trust were predicted by the four cultural dimensions of HI, VI, HC, and VC across countries. Self-conscious emotions and trust, in turn, predicted compliance, intentions to report the infection to health authorities and acquaintances/friends. Gender, age, concern about the spread of the virus and political orientation were added as covariates to the model.

First, we estimated the latent means using the marker method (Little et al., 2006). We fixed the intercept of each factor’s marking item to zero in order to estimate the latent means and interpret them using the same scale as the marker items. Next, we compared means across countries. Finally, we proceeded to examine differences across countries in the model structural paths using the scaled difference Chi-square tests (Satorra and Bentler, 2001).

## Results

### Measurement Invariance

The configural model of the HVIC scale had good fit, χ^2^ (144, *N* = 1896) = 394.34, *p* < 0.001, CFI = 0.95, AGFI = 0.99, RMSEA = 0.05, SRMR = 0.05. Constraining the loadings to be equal across groups did not cause the model to deteriorate significantly, indicating that metric invariance was achieved across countries, ΔCFI = 0.005 (Cheung and Rensvold, 2002). However, fixing intercepts across countries caused the model fit to deteriorate more than the recommended threshold (ΔCFI = 0.04), suggesting that the model did not achieve scalar invariance. Thus, we sought to establish partial scalar invariance by examining what intercepts were the source of the misfit in the model. Releasing the intercepts of two items (“I enjoy working in situations involving competition with others” and “I usually sacrifice my self-interest for the benefit of my group”) to vary freely across countries resulted in a model with partial scalar invariance (ΔCFI = 0.009).

Analogously, we tested for invariance across countries for the compliance measure. The six-item scale demonstrated acceptable fit across groups, χ^2^ (24, *N* = 1896) = 59.557, *p* < 0.001, CFI = 0.98, AGFI = 0.99, RMSEA = 0.07, SRMR = 0.03. The model’s fit did not deteriorate significantly when we constrained intercepts to be equal across countries (ΔCFI = 0.009). However, the model did not achieve full scalar invariance (ΔCFI = 0.029). Therefore, we established partial scalar invariance by releasing the intercepts of the items, “I wash my hands often” and “I stand away from others in public places.”

### Primary Analyses: Comparisons Across Countries

Zero-order correlations, means, standard deviations, and alpha coefficients for measures across countries are summarized in Table 1. The indices of AGFI = 0.99, RMSEA = 0.05, SRMR = 0.06 indicated good fit. CFI was below the recommended threshold of acceptability (=0.91) and χ^2^ (806, *N* = 1896) = 1794.92, *p* < 0.001 was significant. Overall, we judged the model fit adequate and retained this model for cross-country comparisons.

**TABLE 1:** Correlations, Means, standard deviations, and Cronbach’s alpha between study variables separately for each cultural group.

Measure	α_tot_	α_US_	α_IT_	α_K__R_	1	2	3	4	5	6	7	8	9	10	11	12	13	14
1. HI	0.77	0.78	0.65	0.73	–													
2. HC	0.77	0.79	0.79	0.71	0.31*** (0.23***) 0.07 (0.24***)	–												
3. VI	0.74	0.78	0.69	0.68	0.09*** (0.11**) 0.25*** (0.23***)	−0.02 (−0.04) −0.13** (0.35***)	–											
4. VC	0.79	0.77	0.74	0.77	−0.14*** (−0.10*) −0.07 (0.04)	0.05* (0.16***) 0.03 (0.35***)	0.26*** (0.19***) 0.14*** (0.34***)	–										
5. Shame	–	–	–	–	−0.20*** (0.03) −0.05 (−0.01)	−0.22*** (−0.09*) −0.15** (0.03)	0.16*** (0.09*) 0.05 (0.08*)	0.26*** (0.16***) 0.07 (0.13**)	–									
6. Guilt	–	–	–	–	−0.18*** (−0.00) −0.04 (−0.02)	−0.15*** (−0.02) −0.06 (0.04)	0.11*** (0.10*) −0.00 (0.02)	0.23*** (0.22***) 0.09* (0.12**)	0.71*** (0.72***) 0.57*** (0.69***)	–								
7. Trust	–	–	–	–	−0.11*** (−0.01) 0.05 (0.07)	−0.01 (−0.03) 0.15*** (0.20***)	0.14*** (0.26***) 0.06 (−0.01)	0.10*** (0.26***) 0.02 (0.02)	0.10*** (0.05) −0.01 (0.02)	0.15*** (0.01) 0.07 (0.09*)	–							
8. Compliance	0.82	0.76	0.69	0.86	0.23*** (0.08) 0.12** (0.11**)	0.39*** (0.36***) 0.26** (0.27***)	−0.06* (−0.11**) 0.05 (0.13***)	−0.05 (0.09*) −0.01 (0.16***)	−0.19*** (−0.04) −0.06 (0.06)	−0.08*** (0.05) 0.06 (0.08*)	0.02 (−0.06) 0.19*** (0.21***)	–						
9. Self-reporting	–	–	–	–	0.08*** (0.11*) 0.01 (0.11*)	0.21*** (0.27***) 0.19*** (0.19***)	0.01 (0.01) 0.04 (0.04)	0.00 (0.12**) −0.02 (0.05)	−0.08*** (−0.02) −0.14** (−0.04)	0.02 (0.05) −0.01 (0.04)	0.19*** (0.09*) 0.16*** (0.14***)	0.32*** (0.35***) 0.24*** (0.34***)	–					
10. Hiding	–	–	–	–	−0.13*** (0.04) −0.02 (−0.01)	−0.27*** (−0.17***) −0.18*** (−0.17***)	0.10*** (0.07) 0.05 (−0.01)	0.16*** (0.06) 0.07 (0.03)	0.38*** (0.31***) 0.29*** (0.21***)	0.28*** (0.25***) 0.17*** (0.15***)	0.04 (0.05) −0.04 (−0.06)	−0.24*** (−0.15***) −0.17*** (−0.10*)	−0.19*** (−0.19***) −0.25*** (−0.15***)	–				
11. Age	–	–	–	–	−0.22*** (−0.04) 0.02 (−0.21***)	−0.10*** (0.04) −0.09* (0.15***)	0.12*** (−0.08) 0.06 (0.14***)	0.29*** (0.03) 0.19*** (0.24***)	0.20*** (−0.22***) −0.03 (0.09*)	0.04 (−0.25***) −0.08* (−0.03)	−0.03 (0.11**) −0.08* (0.02)	−0.08** (0.08*) 0.07 (0.16***)	−0.08*** (0.06) 0.07 (−0.05)	0.14*** (−0.12**) −0.03 (0.04)	–			
12. Gender	–	–	–	–	0.04 (0.04) −0.02 (0.08*)	0.10*** (0.23***) 0.15*** (−0.02)	−0.21*** (−0.24***) −0.27*** (−0.13**)	−0.11*** (−0.06) −0.08* (−0.19***)	0.00 (0.01) 0.00 (0.01)	0.05* (0.02) 0.07 (0.08*)	−0.01 (−0.08*) 0.02 (0.06)	0.14*** (0.17***) 0.15*** (0.14***)	0.05 (0.06) 0.01 (0.01)	−0.00 (−0.04) −0.03 (0.04)	−0.00 (0.12**) −0.02 (−0.09*)	–		
13. PD	–	–	–	–	0.03 (−0.02) −0.01 (0.05)	0.20*** (0.30***) 0.16*** (0.22***)	−0.02 (0.14***) −0.07 (0.17***)	0.13*** (0.16***) 0.07 (0.12**)	0.12*** (0.11**) 0.31*** (0.18***)	0.17*** (0.18***) 0.04 (0.20***)	−0.17*** (−0.17***) 0.20*** (0.00)	0.36*** (0.52***) 0.16*** (0.12**)	0.11*** (0.23***) 0.34*** (0.10*)	−0.05* (−0.13**) −0.07 (−0.03)	0.15*** (0.14**) 0.10* (0.08*)	0.14*** (0.17***) 0.08* (0.08*)	–	
14. PO	–	–	–	–	−0.12*** (0.00) 0.08* (−0.08*)	−0.20*** (−0.12**) −0.22*** (0.02)	0.34*** (0.31***) 0.34*** (0.23***)	0.25*** (0.20***) 0.17*** (0.14***)	0.20*** (0.07) −0.01 (0.10**)	0.09*** (−0.30) −0.07 (0.05)	0.16*** (0.52***) −0.18*** (−0.19***)	−0.22*** (−0.19***) −0.10* (−0.06)	−0.07** (−0.03) −0.01 (−0.10**)	0.19*** (0.09*) 0.07 (0.12**)	0.25*** (0.13**) 0.16*** (0.15***)	−0.12*** (−0.15***) −0.19*** (−0.02)	−0.05* (−0.24***) −0.05 (0.14***)	–
*M*_tot_ (SD)	–	–	–	–	5.08 (1.14)	5.68 (0.91)	4.34 (1.29)	3.84 (1.24)	2.68 (1.89)	3.20 (1.99)	4.71 (1.76)	6.09 (0.84)	6.14 (1.48)	1.95 (1.56)	37.25 (13.82)	1.52 (0.55)	5.69 (1.42)	42.26 (25.12)
*M*_US__A_ (SD)	–	–	–	–	5.56 (0.91)	5.95 (0.79)	4.17 (1.36)	3.84 (1.29)	2.18 (1.64)	2.53 (1.85)	3.43 (1.81)	6.27 (0.78)	5.78 (1.74)	1.70 (1.44)	39.35 (13.16)	1.53 (0.55)	5.99 (1.32)	38.39 (29.16)
*M*_IT_ (SD)	–	–	–	–	5.31 (0.96)	5.92 (0.81)	4.11 (1.40)	3.29 (1.19)	1.77 (1.28)	2.75 (1.83)	5.32 (1.38)	6.39 (0.61)	6.61 (0.93)	1.40 (0.97)	26.94 (7.72)	1.53 (0.55)	5.37 (1.54)	35.10 (22.75)
*M*_KOR_ (SD)	–	–	–	–	4.46 (1.19)	5.23 (0.92)	4.68 (1.04)	4.33 (1.02)	3.92 (1.88)	4.18 (1.85)	5.27 (1.41)	5.68 (0.91)	6.04 (1.52)	2.65 (1.81)	44.46 (13.15)	1.52 (0.54)	5.68 (1.35)	51.85 (19.83)

Correlations between variables for the entire sample (*N* = 1896), American (*N* = 597), Italian (*N* = 606), and Korean (*N* = 693) participants are presented in order. Correlations for American and Korean participant are presented in parenthesis. For all scales, higher scores are indicative of more extreme responding in the direction of the construct assessed. HI, horizontal individualism; VI, vertical individualism; HC, horizontal collectivism; VC, vertical collectivism; PD, perceived danger; PO, political orientation; self-reporting, intentions to report contracting the disease to health authorities; hiding, intentions to hide contracting the infection from acquaintances and friends. ***p < 0.001, **p < 0.01, *p < 0.05.

#### Latent Means

The measurement models of the latent variables were constrained to be partially invariant across countries to enable comparisons of latent means across groups. Results of latent means comparisons using the market method are summarized in Table 2. Participants in the United States endorsed HI and VI significantly more strongly than participants in Korea. Italian participants did not differ significantly from those in the United States and Korea in their endorsement of HI and VI but endorsed HC significantly more strongly than participants in either country. Participants in Korea endorsed VC more strongly than Italian or United States participants. Moreover, participants reported stronger compliance with social distancing norms in Italy, followed by the United States and then Korea.

**TABLE 2:** Latent means and standard errors in the structural equation model.

	United States (SE)	Italy (SE)	South Korea (SE)
Horizontal individualism	5.41^*a*^ (0.26)	5.09^*a,b*^ (0.26)	4.62^*b*^ (0.31)
Horizontal collectivism	4.77^*a*^ (0.26)	5.87^*b*^ (0.19)	4.28^*a*^ (0.25)
Vertical individualism	4.36^*a*^ (0.28)	3.85^*a,b*^ (0.24)	3.38^*b*^ (0.20)
Vertical collectivism	2.11^*a*^ (0.34)	1.70^*a*^ (0.29)	3.57^*b*^ (0.26)
Compliance	4.76^*a*^ (0.28)	5.82^*b*^ (0.17)	2.94^*c*^ (0.29)

*Means that do not share a superscript within each row are significantly different at *p* ≤ 0.05. Means were estimated using the marker method and are therefore interpretable on a 1 to 7 scale.*

#### Structural Paths

Fixing all the structural paths (except the covariates) to be the same across countries produced a significantly worse fit, Δ*χ*^2^(42) = 67.954, *p* = 0.006. This result suggested the presence of differences between some of the paths. To examine these differences, we proceeded by systematically constraining one path at the time to be equal across countries. Fixing the effects of the HI, HC, VI and VC paths on shame [*Δ*χ*^2^(2) < 5.57*, *p* > 0.06] or guilt [*Δ*χ*^2^(2) < 4.91*, *p* > 0.08] across countries did not produce a significantly worse fit. Similarly, fixing the effects of HI and VC on trust did not worsen the fit significantly [*Δ*χ*^2^(2) < 4.04*, *p* > 0.13], but fixing either paths between HC [*Δ*χ*^2^(2) < 7.64*, *p* = 0.02] or VC [*Δ*χ*^2^(2) < 12.14*, *p* = 0.002] and trust produced a significantly worse fit. Thus, these paths were allowed to vary freely.

**FIGURE 1:**
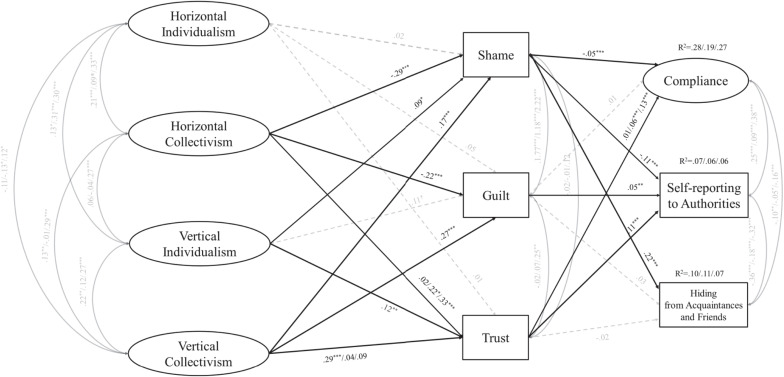
Structural equation model showing unstandardized coefficients. Multiple coefficients indicate an unconstrained path and are reported as USA/IT/KOR. Dashed lines are non-significant paths. Gender, age, perceived concern, and political orientation are covariates in the model. ***p < 0.001, **p < 0.01, *p < 0.05, ^‡^ < 0.10.

Next, fixing the paths between shame [*Δ*χ*^2^(2) < 2.96*, *p* > 0.22] or guilt [*Δ*χ*^2^(2) < 0.56*, *p* > 0.75] and compliance, intentions to report the infection to authorities, or acquaintances/friends did not produce a significantly worse fit. Only the path between trust and compliance [*Δ*χ*^2^(2) < 16.17*, *p* < 0.001] was allowed to vary freely across countries, whereas the path between trust and intentions to report the infection to authorities or acquaintances/friends [ *Δ*χ*^2^(2) < 2.43*, *p* > 0.29] were fixed. The resulting model with the freed paths specified above had no significantly worse fit than the model where all paths were free to covary [*Δ*χ*^2^(36) < 36.43*, *p* = 0.454; χ^2^ (842, *N* = 1896) = 1834.27, *p* < 0.001, AGFI = 0.99, RMSEA = 0.05, SRMR = 0.06]. The model unstandardized solution is comparable across groups (Kline, 2015) and is summarized in Figure 1. Within groups completely standardized solutions for each country are reported in Table 3.

**TABLE 3:** Within groups completely standardized solutions for coefficients in the study.

	United States		Italy		South Korea			United States		Italy		South Korea	
Paths	β	SE	β	SE	β	SE	Paths	β	SE	β	SE	β	SE
HC → shame ^=^	−0.13***	0.07	−0.17***	0.07	−0.14***	0.07	Shame→ compliance ^=^	−0.11***	0.03	−0.14***	0.01	−0.10***	0.01
HI → shame ^=^	0.01	0.05	0.01	0.05	0.01	0.05	Guilt→ compliance ^=^	0.05	0.02	0.03	0.02	0.01	0.01
VI → shame ^=^	0.06*	0.04	0.08*	0.04	0.04*	0.04	Trust→ compliance ^=^	0.12	0.02	0.19***	0.02	0.18***	0.03
VC → shame ^=^	0.13***	0.04	0.15***	0.04	0.09***	0.04	Shame→ self−reporting ^=^	−0.11***	0.03	−0.16***	0.03	−0.14***	0.03
HC → guilt ^=^	−0.09***	0.07	−0.09***	0.07	−0.11***	0.07	Guilt → self−reporting ^=^	0.05**	0.02	0.10**	0.02	0.06**	0.02
HI → guilt ^=^	−0.03	0.06	−0.03	0.06	−0.03	0.06	Trust→ self−reporting ^=^	0.12***	0.02	0.17***	0.02	0.10***	0.02
VI → guilt ^=^	0.07	0.06	0.07	0.06	0.05	0.06	Shame→ hiding ^=^	0.26***	0.03	0.29***	0.03	0.23***	0.03
VC → guilt ^=^	0.19***	0.05	0.17***	0.05	0.14***	0.05	Guilt→ hiding ^=^	0.04	0.02	0.05	0.02	0.03	0.02
HC → trust ^=^	0.01	0.12	0.12*	0.10	0.20***	0.09	Trust→ hiding ^=^	−0.03	0.02	−0.03	0.02	−0.02	0.02
HI → trust ^=^	0.001	0.05	0.001	0.05	0.01	0.05							
VI → trust ^=^	0.08**	0.05	0.10**	0.05	0.07**	0.05							
VC → trust ^=^	0.21***	0.07	0.04	0.06	−0.06	0.08							

HI, horizontal individualism; HC, horizontal collectivism; VI, vertical individualism; VC, vertical collectivism; self-reporting, intentions to disclose contracting the disease to health authorities; hiding, intentions to hide contracting the disease from acquaintances and friends; ^=^, indicates structural paths constrained to be invariant across groups; ***p < 0.001, **p < 0.01, *p < 0.05.

Guilt and shame were negatively predicted by HC and positively predicted by VC across countries. Interestingly, HC significantly and positively predicted trust only in Italy (*b* = 0.22, *p* = 0.02) and Korea (*b* = 0.33, *p* < 0.001). In the United States, trust was predicted by VC (*b* = 0.29, *p* < 0.001). There were some smaller but significant associations between VI and shame (*b* = 0.09, *p* = 0.01), and VI and trust (*b* = 0.12, *p* = 0.04). There was no significant association between HI and other constructs, but only a marginally significant association (*p* = 0.054) with trust.

Shame negatively predicted compliance and individuals’ intentions to report the infection to authorities and positively predicted individuals’ intentions to hide the disease from acquaintances/friends. Conversely, stronger guilt was positively associated with individuals’ intentions to report the disease to authorities. Finally, stronger trust positively predicted individuals’ intentions to report the infection to authorities. However, trust was significantly associated with compliance only in Italy (*b* = 0.06, *p* < 0.001) and Korea (*b* = 0.13, *p* < 0.001).

We inspected the indirect effects from the cultural orientations of HI, VI, HC, and VC to the criterion variables via self-conscious emotions and trust. The indirect effects of HC via shame on compliance, *b* = 0.02, SE = 0.01, CI_95%_ (0.004 to 0.03), self-reporting to health authorities, *b* = 0.03, SE = 0.01, CI_95%_ (0.01 to 0.06), and hiding from acquaintances/friends, *b* = −0.07, SE = 0.02, CI_95%_ (−0.10 to −0.03) were significant. HC was also indirectly (and negatively) associated with self-reporting via guilt *b* = −0.01, SE = 0.01, CI_95%_ (−0.02 to −0.001]. The indirect effects of VC via shame on compliance, *b* = −0.01, SE = 0.003, CI_95%_ (−0.014 to −0.002), self-reporting, *b* = −0.02, SE = 0.01, CI_95%_ (−0.03 to −0.01), and hiding, *b* = 0.04, SE = 0.01, CI_95%_ (0.02 to 0.06), were also significant (and in the opposite directions, compared to HC). There was a positive indirect effect of VC via guilt on self-reporting, *b* = 0.01, SE = 0.01, CI_95%_ (0.003 to 0.03). None of the indirect effects of VI were significant.

Finally, with regard to trust, the indirect effects of HC via trust on compliance was significant in the Italian *b* = 0.02, SE = 0.01, CI_95%_ (0.01 to 0.03) and Korean samples, *b* = 0.04, SE = 0.01, CI_95%_ (0.01 to 0.07). The indirect effect of HC on self-reporting via trust was significant only in the Korean sample *b* = 0.04, SE = 0.01, CI_95%_ (0.01 to 0.06). In the United States, the indirect effect of VC on self-reporting via trust was significant, *b* = 0.03, SE = 0.01, CI_95%_ (0.01 to 0.05). Other indirect effects were not statistically significant.

## Discussion

In this research, we investigated how trust in government and self-conscious emotions of shame and guilt explained individuals’ compliance with social distancing, and their intentions to report the infection to health authorities or acquaintances/friends. These associations were investigated in three countries characterized by different cultural themes (the United States, Italy, and Korea). In each country, we also measured individuals’ cultural orientations. Results indicated the existence of cultural similarities across contexts. Differences mostly emerged with regard to trust.

In all three countries, feelings of shame at the idea of contracting the virus were negatively associated with compliance and individuals’ self-reporting intentions. These associations emerged regardless of the overall cultural theme of the country, suggesting that they are stable across cultures. The findings suggest that stigmatizing or blaming individuals for contracting the infection could potentially backfire.

Several recent episodes reported in the news or on social media imply the existence of stigmatizing attitudes toward people who are perceived as flaunting lockdown rules and social distancing norms. For instance, hashtags such as “covidiots” (a portmanteau combining the words “covid-19” and “idiots”) are used on Twitter to mock or blame individuals who do not abide by the norms (Reicher and Drury, 2021). Moreover, there are anecdotical reports of people being attacked, insulted or publicly shamed because they were found walking in the streets during the lockdown. The president of an Italian region even asked for lists of violators to be made public, intending to shame transgressors (Pellegrino, 2020). Such discourses and actions are unlikely to have positive implications for individuals’ willingness to abide by the new norms. Instead, feelings of shame could even limit the authorities’ ability to trace and test new cases, or acquaintances’ and friends’ ability to know about potential contacts with infected individuals.

Results about shame were obtained controlling for the effects of guilt. Conversely, the only effect of guilt independent of shame was a positive association between guilt and individuals’ intentions to report the infection to authorities. This finding is congruent with previous research suggesting that guilt may foster more constructive responses to transgressions (Tangney and Fischer, 1995). Notably, however, guilt explained less overall variance compared to shame.

Finally, the hypothesized association between trust in government and compliance was statistically significant only in Italy and South Korea. In contrast, the association in the United States was closer to zero and non-statistically significant. Although we controlled for participants’ political orientation, this pattern of associations could reflect the political situation in the United States at the time of the study, where the Republican-led central government had been notably slow in its responses to the pandemic, sending out contradictory signals to the public and undermining the experts’ recommendations (cf. Mirvis, 2020). Nonetheless, the association between trust and self-reporting did not differ significantly across countries, indicating that individuals who trust the government’s handling of the pandemic are more likely to report the infection to authorities.

### Cultural Orientations and Cross-Cultural Comparisons

The pattern of means of the cultural orientations of HI, HC, VI, and VC suggested that overall levels of individualism were higher in the United States than Korea and that the Italian sample fell between these two countries. Whereas Korean participants reported higher levels of VC than other groups, Italian participants reported higher HC levels. Within countries, however, there were strong similarities concerning the role of cultural orientations.

Vertical collectivism (and to a lesser extent VI) was positively associated with individuals’ feelings of shame at the thought of contracting the disease. This resulted in a negative indirect effect of VC on compliance and intentions to report the infection to authorities and acquaintances/friends. Conversely, HC was negatively associated with shame. HC may be less conducive to stigma concerning the infection, thus creating a positive indirect effect on compliance and self-reporting intentions. There were also significant associations between VC, HC and guilt. Individuals who endorsed VC were also more likely to report stronger guilt concerning the infection.

Conversely, those who endorsed HC reported lower feelings of guilt. This pattern of associations created contrasting indirect effects of VC and HC on individuals’ intentions to report the disease to authorities. Specifically, the indirect effect of HC on self-reporting intentions via guilt was negative, whereas that of VC was positive.

The findings highlight the relevance of individuals’ cultural orientations in their responses to the pandemic. Across countries, participants who valued vertical relationships were more likely to perceive stronger self-conscious emotions. Instead, valuing horizontal relationships was associated with weaker self-conscious emotions. These emotions, in turn, predicted different responses to the disease. Shame was associated with less constructive (from the perspective of the group) responses, whereas guilt was linked to a higher likelihood of reporting the infection to authorities. The values of individualism (whether horizontal or vertical) were overall less relevant, and HI and VI’s indirect effects were non-statistically different from zero.

The larger difference across countries concerned the articulation between cultural orientations, trust and individuals’ responses to the virus. Research on the association between cultural orientations and trust indicates the existence of a complex and multifaceted relationship among these constructs (e.g., Shin and Park, 2004; Realo and Allik, 2009). Our results indicated that in Italy and South Korea, individuals who endorsed the HC orientations were also more likely to trust the governments. This finding is in line with previous research emphasizing the importance of values of interdependence in predicting generalized trust (Shin and Park, 2004). This finding also highlights the fact that trust in government does not depend only on government performance (Keele, 2007), which likely varied across countries. Rather, our findings highlight the relevance of valuing cohesive relationships for individuals’ trust toward the government (Job, 2005). Consistent with this idea, there was an indirect effect of HC on compliance via trust in both Italy and Korea. HC’s indirect effect on self-reporting intentions was instead significant only in Korea, a result likely due to the stronger association between HC and trust in this country.

Differently from Italy and South Korea, VC predicted stronger trust in the government in the United States. Trust in government’s action was not significantly associated with greater compliance with social distancing norms in that country. At the time of the study, the federal administration had emphasized the importance of loyalty, deference to authority and an “America first” policy. It has also repeatedly signaled its contempt for scientific advice or social distancing norms. For instance, President Trump stated (via social networks) his support for protesters who openly defied lockdown orders in Michigan or elsewhere. This political response might explain why VC values predicted individuals’ trust for the government in the United States. Notably, however, the association between VC and trust was significant independently from individuals’ political orientation or concern for the spread of the coronavirus within the country, underlining cultural orientations’ relevance *vis-à-vis* trust.

## Limitations, Future Directions, and Conclusion

This research is the first to report evidence for the roles of self-conscious emotions and trust in individuals’ compliance and self-reporting intentions. We complemented current work on the pandemic by examining the relationships between cultural orientations and these constructs across three countries (the United States, Italy and South Korea). Nonetheless, the research was affected by some limitations.

First, some constructs in the study, such as trust in the government and self-conscious emotions, were measured with single-item measures. The use of single-item measures offers practical advantages, such as reducing the survey’s completion time and minimizing participants’ drop-outs. Notably, single-item measures tend to have similar predictive validity to multiple-item measures when they tap constructs that are “singular” and can be “concretely imagined” by participants (Rossiter, 2002; Bergkvist and Rossiter, 2007). However, future research may consider the use of multiple-item measures, which enable researchers to examine the role of different facets of a construct (Churchill, 1979).

Another limitation of this study concerns the fact that some emotions relevant in the context of individuals’ reactions to the pandemic were not measured. For example, it is likely that individuals’ feelings of fear could play a role in their intentions to comply with social distancing, or report the disease to the authorities (e.g., Harper et al., 2020). Moreover, feelings of disgust may augment the sense of stigma or the stigmatization of those who have contracted the infection (e.g., Herek et al., 2002). A priority for future work is to consider the role of these, and other emotional reactions, in the context of the current pandemic, as well as cross-cultural similarities and differences in people’s appraisals of the infection.

Finally, it should be noted that individualism and collectivism are complex “syndromes,” encompassing a cluster of different beliefs, norms and practices as well as emphasizing different levels of self-construal (see Brewer and Chen, 2007). Whereas individualism has generally been associated with an independent self-construal, collectivism has been linked to an interdependent self-construal (Markus and Kitayama, 1991). Nonetheless, more recent work highlights that the relationship between individualism-collectivism and self-construal is multifaceted (Vignoles et al., 2006). A given cultural context may foster independence in a particular domain of the self, but interdependence in another domain. Thus, there are multiple different ways of being independent and interdependent. Future research should examine how different configurations of independence and interdependence are associated with trust in government and, indirectly, compliance with social distancing and self-reporting intentions.

To conclude, our findings indicate that attempting to deter people from defying social distancing by blaming or stigmatizing them may negatively impact public health. Results about guilt had slightly more positive implications. However, it is hard to separate feelings of guilt from those of shame, especially in some cultures (Wong and Tsai, 2007). Thus, governments and decision-makers may obtain better results by focusing on the importance of social cohesion and trustworthiness in their attempts to tackle the pandemic and manage public responses.

## Data Availability Statement

The raw data supporting the conclusions of this article will be made available by the authors, without undue reservation.

## Ethics Statement

The studies involving human participants were reviewed and approved by the Psychology Ethics Committee of Leeds Beckett University. The participants provided their informed consent online prior to taking part in this study.

## Author Contributions

GT and CM: manuscript writing, editing, and data analysis. Both authors contributed to the article and approved the submitted version.

## Conflict of Interest

The authors declare that the research was conducted in the absence of any commercial or financial relationships that could be construed as a potential conflict of interest.

## Footnotes

1 Participants were paid in USD, EUR, and WON, respectively. Here we report prices converted in GBP.

2 In the present study, we did not include the two items “My happiness depends very much on the happiness of those around me” and “Children should feel honored if their parents receive a distinguished award” from Sivadas et al.’s (2008) scale because these items had inconsistent loadings in previous cross-cultural research involving samples from the United States and Korea (Moon et al., 2018).
